# Responsiveness and Minimal Important Change of the Arabic Disabilities of the Arm, Shoulder and Hand (DASH) in Patients with Upper Extremity Musculoskeletal Disorders

**DOI:** 10.3390/healthcare11192623

**Published:** 2023-09-26

**Authors:** Ali H. Alnahdi

**Affiliations:** Department of Rehabilitation Sciences, College of Applied Medical Sciences, King Saud University, P.O. Box 10219, Riyadh 11433, Saudi Arabia; alialnahdi@ksu.edu.sa; Tel.: +966-14693595; Fax: +966-14693589

**Keywords:** upper limb, psychometrics, activity limitation, minimal clinically important change, longitudinal construct validity

## Abstract

The aim of this study was to examine the responsiveness of the Arabic Disabilities of the Arm, Shoulder and Hand (DASH) and to quantify its minimal important change (MIC) for improvement. People with upper extremity musculoskeletal problems who were receiving physical therapy were evaluated at baseline and again during a follow-up appointment, with a median time frame of 7 days between the two testing sessions (range of 6 to 72 days). The participants completed the Arabic DASH, Global Assessment of Function (GAF), Numeric Pain Rating Scale (NPRS) and Global Rating of Change Scale (GRC). The responsiveness of the Arabic DASH was assessed by examining the pre-specified hypotheses. The MIC for improvement was determined using the receiver operating characteristic method (MIC_ROC_) and the predictive modeling method (MIC_pred_). As hypothesized, a change in the Arabic DASH demonstrated a significant positive correlation with changes in the GAF (r = 0.69), NPRS (r = 0.68) and GRC (r = 0.73). Consistent with our hypotheses, the DASH change scores could be used to differentiate between participants who improved and those who did not improve (area under the receiver operating characteristic curve = 0.87), and they showed a large magnitude of change (effect size = 1.53, standardized response mean = 1.42) in patients who improved. All the hypotheses specified a priori were supported by the results. The Arabic DASH MIC_ROC_ and MIC_pred_ were estimated to be 14.22 and 14.85. The interaction between the DASH change and baseline score was not a significant predictor of status (improved vs. not improved) (*p* = 0.75), indicating that the DASH MIC was not baseline-dependent. The Arabic DASH demonstrated sufficient responsiveness, supporting the idea that the Arabic DASH is capable of detecting changes in upper extremity function over time. The value of the Arabic DASH MIC was similar when estimated using the predictive modeling and ROC methods, and the MIC was not dependent on baseline status.

## 1. Introduction

The Disabilities of the Arm, Shoulder and Hand (DASH) is a commonly used patient-reported outcome measure (PROM) to measure upper extremity function and symptoms [1,2]. Recent systematic reviews of translated versions of the DASH indicated that most translated versions underwent only partial evaluation of psychometric properties and that the majority of studies that examined the psychometric properties of these translated versions had a poor methodological quality [3,4,5]. These reviews point to the need for a rigorous methodology and further assessments of translated DASH psychometric properties, especially responsiveness and interpretability.

Responsiveness refers to the ability of a PROM to detect changes over time in the construct measured [6]. Hypothesis testing is the recommended method for assessing responsiveness according to the consensus-based standards for the selection of health measurement instruments (COSMIN) guidelines [7,8]. A large number of studies that examined the responsiveness of the DASH and its translated versions lack specific hypotheses to examine whether the DASH change scores actually reflect changes in upper extremity function [9,10,11,12,13,14,15,16]. Currently, there is only one study that reported an assessment of the responsiveness of the Arabic version of the DASH [9]. In this study, the authors used effect size indices and the area under the receiver operating characteristic curve to examine the responsiveness. This study lacked any specific pre-defined hypotheses regarding DASH change scores, and the use of effect size indices is inappropriate when examining responsiveness based on COSMIN [7,8].

A PROM’s minimal important change (MIC) is defined as “the smallest change in score in the construct to be measured which patients perceive as important” [7,17]. Determining the MIC is not part of responsiveness testing but rather is related to the interpretability of PROM change scores [7]. The MIC can be determined using distribution- or anchor-based methods, with the latter believed to be superior [17,18,19]. Numerous studies determined the MIC for various translated versions of the DASH using the receiver operating characteristic (ROC), which is an anchor-based method [9,10,20,21,22,23,24,25]. Recently, a predictive modeling method, which is also an anchor-based method, has been reported to be more precise compared to the commonly used ROC method [26]. Additionally, the majority of studies that estimated the DASH MIC, including the Arabic DASH, had unbalanced proportions of participants who improved and those who did not, which is known to bias the MIC estimate [27].

To date, no prior study has examined the responsiveness of the Arabic DASH using the recommended method of hypothesis testing, and no study has determined the Arabic DASH MIC using the more precise predictive modeling method. Thus, the aims of the current study were (1) to examine the responsiveness of the Arabic DASH in patients with upper extremity musculoskeletal disorders and (2) to quantify the MIC for improvement in the Arabic DASH and to examine whether the MIC value is dependent on baseline status. We hypothesized that the Arabic DASH would have sufficient responsiveness in detecting changes over time in upper extremity function.

## 2. Materials and Methods

### 2.1. Setting and Participants

The participants in the current study were recruited using convenience sampling from four outpatient physical therapy departments (King Saud University Medical City, Security Forces Hospital, King Abdulaziz Medical City and Physiotri Clinic) in Riyadh, Saudi Arabia. Approval to conduct the study was obtained from the Institutional Review Boards of King Saud University Medical City (18/0372/IRB) and Security Forces Hospital (H-01-R-069). The purpose and procedure of the study were explained to all participants, and signed informed consent forms were obtained before participation. The inclusion criteria used in the current study were (1) primary complaint of upper extremity musculoskeletal disorder and (2) ≥18 years of age. The exclusion criteria used in the current study were (1) not able to read and understand the Arabic language and (2) neurological, cardiovascular, pulmonary or spinal disorders reported by the participant as a cause of functional limitation. In the current study, we aimed to include participants in which the major cause of their functional limitation was upper extremity musculoskeletal disorder. Thus, participants with additional concomitant disorders (neurological, cardiovascular, pulmonary or spinal disorders) were excluded only if these disorders were perceived by the patients to cause functional limitation.

### 2.2. Procedure

The current study was a prospective cohort study with two assessment time points (baseline and follow-up assessments). The baseline assessment was conducted during the first visit of the participant to the outpatient physical therapy department, while the follow-up assessment was conducted during a follow-up visit. Between the baseline and follow-up assessments, participants were under physical therapy care, which was the responsibility of the treating physical therapist. Given that the current study was an observational study, the type and specifics of the physical therapy interventions were not determined or controlled by the research team. The physical therapy treatment provided included but was not limited to patient education, therapeutic exercises, therapeutic modalities, articular and soft tissue manual therapy, taping and dry needling. The research team had no involvement in the type or details of the physical therapy care provided to the participants. The participants completed the Arabic DASH [9], Numeric Pain Rating Scale [28] and Global Assessment of Function [29,30] during the baseline assessment. During the follow-up assessment, the participants completed the same outcome measures, in addition to the Global Rating of Change Scale [31,32].

### 2.3. Outcome Measures

#### 2.3.1. Disabilities of the Arm, Shoulder and Hand (DASH)

The DASH is a PROM designed to quantify upper extremity function and symptoms using 30 items [1,2]. The scoring of the DASH items ranged from no functional limitation and no symptoms (score of 1) to functional inability and extreme symptoms (score of 5). The DASH was reported using a total score from 0 to 100, with a higher score indicating the worst function and symptoms. A prior research report supported the validity and reliability of the Arabic version of the DASH [9].

#### 2.3.2. Numeric Pain Rating Scale (NPRS)

The NPRS was used to measure the average pain intensity at the site of the upper extremity dysfunction [33]. The NPRS score ranged from no pain (score of 0) to worst pain imaginable (score of 10) [33]. Prior research studies supported the measurement properties of the Arabic version of the NPRS [28,30].

#### 2.3.3. Global Assessment of Function (GAF)

The GAF is a self-reported outcome used to measure participants’ overall functional ability [30,34]. The GAF score ranged from inability to perform any activity of daily living (score of 0) to ability to perform all activities of daily living without difficulty (score of 100) [30,34]. The measurement properties of the GAF have been supported by prior research studies [30,34].

#### 2.3.4. Global Rating of Change Scale (GRC)

The participants rated their perceived change in upper extremity function during the second assessment relative to the baseline assessment using the GRC. The GRC score ranged from a very great deal worse (score of −5) to a very great deal better (score of 5) [30,32,34]. The GRC was used to stratify participants into those with improved upper extremity function (GRC of 3 “moderately better”, 4 “great deal better” or 5 “very great deal better”) and those with no improvement (GRC ≤ 2 “little bit better”).

#### 2.3.5. Statistical Analysis

Five pre-defined hypotheses were used to examine the responsiveness of the Arabic DASH [17,35] (Table 1). These hypotheses were formulated based on the argument that the DASH is a measure of upper extremity function and symptoms and that changes in DASH scores reflect changes in the same constructs. In the current study, the Arabic DASH was deemed to have sufficient responsiveness if at least 4 out of the 5 hypotheses were supported, surpassing the recommended threshold of 75% [8,36]. Pearson’s and Spearman’s correlation coefficients were used to examine the hypothesized correlation between changes in the Arabic DASH and changes in the other measures (Table 1). The use of either Pearson’s or Spearman’s correlation coefficients was determined after assessing data conformation to a normality assumption using histograms visual inspection and the Shapiro–Wilk test. In the current study, positive change scores indicated improvement, while negative change scores indicated worsening. Multiple dependent *t*-tests (with no correction for possible inflation of type 1 error) were used to examine the differences in DASH, NPRS and GAF scores between the baseline and follow-up assessments. The effect size (ES) was computed using the mean change divided by the standard deviation of the baseline score, while the standardized response mean (SRM) was computed using the mean change divided by the standard deviation of the change score [37]. ES and SRM were used as part of hypothesis testing and were also used to quantify the magnitude of change observed in the PROM following intervention. ES and SRM values of 0.20, 0.50 and 0.80 were interpreted as small, medium and large effect sizes [38].

To enhance the interpretability of Arabic DASH change scores, the Arabic DASH MIC for improvement was determined using the predictive modeling method (MIC_pred_) and the receiver operating characteristic curve (ROC) method (MIC_ROC_) [26,39]. Logistic regression analysis was used to determine the MIC_pred_ with the GRC anchor (improved vs. not improved) as the dependent variable and the DASH change score as the independent variable. After running the logistic regression analysis, the MIC_pred_ was determined using the following equation: MIC_pred_ = (ln(Odds_pre_) − C)/B, where ln(Odds_pre_) is a natural logarithm for the odds of improvement before knowing the DASH change score, C is the logistic regression intercept and B is the logistic regression coefficient [26]. To account for the possible influence of patients’ baseline status on the value of the MIC, the predictive modeling method was used to determine the MIC_pred_ for patients with a low baseline severity and those with a high baseline severity [26]. The predictive modeling method enables the computation of the MIC_pred_ for both subgroups using the data of the whole sample without dividing the sample into subgroups. The baseline median NPRS score was used to stratify the participants into low vs. high baseline severity to avoid the reported bias in determining the MIC when the baseline score of the measure of interest, DASH, is used for stratification [40]. To determine whether baseline severity influenced the MIC_pred_, a logistic regression model was used to predict the GRC anchor (improved vs. not improved) which included the DASH change score, baseline score and interaction between the DASH change score and baseline score. A significant interaction between the DASH change score and baseline score was used to indicate the baseline dependency of the DASH MIC_pred_.

The MIC_ROC_ was determined to be the optimal DASH change score cutoff point in the ROC curve, best discriminating between participants who improved and those who did not improve and providing the best balance between specificity and sensitivity with the least misclassifications ((1 − specificity) + (1 − sensitivity)) [17]. The ROC curve was created by graphing the rate of false positive on the horizontal axis and the rate of true positive on the vertical axis, using various DASH change score thresholds. The ROC curve was also used in the current study to examine the responsiveness of the DASH. An area under the curve (AUC) of at least 0.70 was used to support the ability of the DASH change score to differentiate between participants with a reported improvement in upper extremity function and those with no improvement (based on the GRC anchor), thus indicating sufficient responsiveness (Table 1) [8,36]. All statistical analyses were conducted using IBM SPSS Statistics 26 (IBM Corp., Armonk, NY, USA) and JASP software (Version 0.17.2).

### 2.4. Sample Size Estimation

A sample size of 100 participants has been recommended to determine the MIC of a patient-reported outcome measure [19]. A sample size of 100 participants is also sufficient to examine the responsiveness of a PROM using hypothesis testing according to the COSMIN recommendations [41]. Based on that, a sample size of 100 was considered sufficient for the purpose of the current study to examine the responsiveness of the Arabic DASH and to determine its MIC value.

## 3. Results

One hundred and fifteen participants were recruited in the current study (Table 2). The site of dysfunction detailed in Table 2 refers to the primary site of upper extremity dysfunction that was the reason for seeking physical therapy care. No interaction was observed in terms of site of dysfunction and all participants presented with only one dysfunction site. The descriptive statistics of the outcome measures in the baseline and follow-up assessments are listed in Table 3. Seventy-three participants missed DASH item 21, two participants missed item 5, and one participant missed items 25, 26, 27 and 28 in the baseline assessment. Seventy-eight participants missed DASH item 21 and one participant missed item 10 in the follow-up assessment. The median time interval between the baseline and follow-up assessments was 7 days (range of 6 to 72 days). Based on the GRC completed during the follow-up assessment, 56 participants reported improved upper extremity function, while 59 participants reported no improvement in upper extremity function (Table 4). No floor or ceiling issues were identified in the DASH scores in both the baseline and follow-up assessments (Table 3).

The participants had a significant improvement in upper extremity function and symptoms, as indicated by the significant reduction in the DASH scores over time (*p* < 0.001) (mean difference: 17.14 points; 95% CI of the difference: 12.57–21.71), the significant increase in the GAF scores over time (*p* < 0.001) (mean difference: 17.14 points; 95% CI of the difference: 13.05–21.23) and the significant reduction in the NPRS scores over time (*p* < 0.001) (mean difference: 1.84 points; 95% CI of the difference: 1.30–2.37) (Table 3).

**Figure 1:**
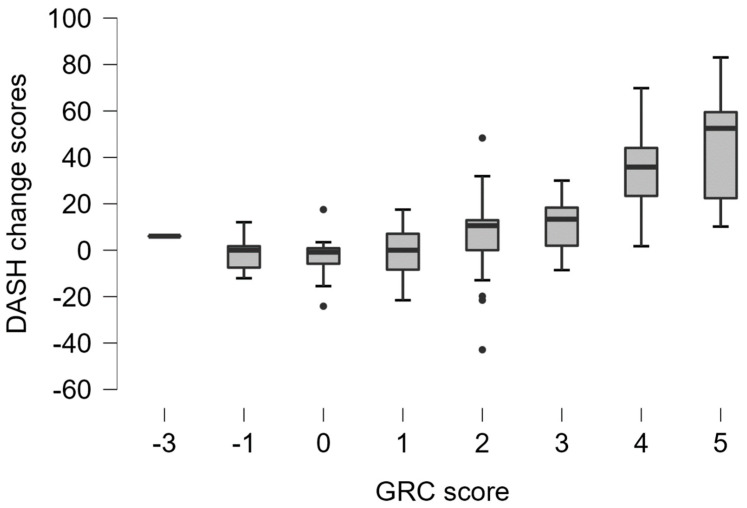
Boxplot of the DASH change scores stratified with the Global Rating of Change (GRC) scores.

**Figure 2:**
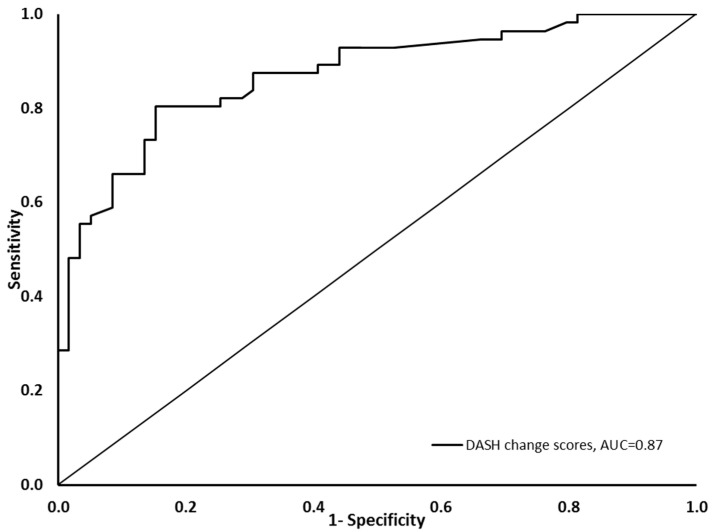
Receiver operating characteristic curve of the DASH change scores. The vertical axis shows the sensitivity, while the horizontal axis shows the 1 − specificity.

The DASH change scores had a significant positive correlation with the GAF and NPRS change scores (Table 5). The DASH change scores also demonstrated a significant positive correlation with the GRC (Table 5) (Figure 1). All of the reported correlations showed point estimates and 95% confidence intervals above the hypothesized levels. The DASH demonstrated a medium to large effect size in the whole sample but a large effect size in participants with a reported improvement in upper extremity function (GRC ≥ 3) (Table 3). The DASH change score demonstrated an AUC of 0.87 (95% CI: 0.81–0.94) (*p* < 0.001) (Figure 2), indicating that the DASH change scores differentiated between participants who improved and those who did not. The MIC_ROC_ for the DASH was 14.22, with a sensitivity of 0.80 and a specificity of 0.85. The MIC_pred_ for the DASH was 14.85 (95% CI: 9.52–20.90). The MIC_pred_ for patients with a low severity was 13.58 (95% CI: 6.391–21.74), while the MIC_pred_ for patients with a high severity was 16.87 (95% CI: 7.01–28.59). The interaction between the DASH change score and baseline score in the logistic regression model was not a significant predictor of patient status (improved vs. not improved) (*p* = 0.75), indicating that the DASH MIC was not dependent on the baseline score.

## 4. Discussion

The aims of the current study were to examine the responsiveness of the Arabic DASH, to quantify the MIC for improvement in the Arabic DASH (using predictive modeling and ROC methods) and to examine whether the MIC value is dependent on patients’ baseline status. The Arabic DASH demonstrated sufficient responsiveness, supporting the idea that the Arabic DASH is capable of detecting changes over time in upper extremity function. The value of the Arabic DASH MIC was similar when estimated using the predictive modeling and ROC methods, and the MIC was not dependent on baseline status.

To the best of our knowledge, this is the first study that examined the responsiveness of the Arabic DASH using hypothesis testing with very specific hypotheses in accordance with the COSMIN guidelines [6]. Five pre-defined hypotheses were formulated for the purpose of assessing the responsiveness of the DASH (Table 1). The responsiveness of the Arabic DASH was determined to be sufficient given that the results supported all five pre-defined hypotheses [8,36].

Positive change scores in the Arabic DASH indicating an improvement in upper extremity function were hypothesized to correlate with positive change scores in the GAF, which also indicate an improvement in functional ability. The findings of the current study supported the direction and magnitude of the hypothesized correlation. During the development of the original DASH, a change in the DASH moderately correlated with a global measure of function (r = 0.69), consistent with the findings of the current study [2]. The same correlation pattern was also reported in other translated versions of the DASH. In the Norwegian version, DASH change scores correlated with changes in the Shoulder Pain and Disability Index function (r = 0.82) and changes in SF-36 physical functioning (r = 0.61) [21]. Similarly, the Dutch DASH change exhibited a moderate correlation with changes in other measures of function, such as Constant–Murley activities of daily living (r = 0.64), SF-36 physical component summary (r = −0.56), SF-36 physical functioning (r = 0.57) and the usual activities domain of the five European quality of life dimensions (r = 0.50) [22].

Given that the DASH represents both upper extremity function and symptoms, positive change scores in the Arabic DASH indicating an improvement in upper extremity function and reduced symptoms were hypothesized to correlate with positive change scores in the NRPS, indicating a reduced pain intensity. The results of the current study supported this hypothesized correlation in terms of direction and magnitude. A correlation between changes in the DASH and changes in pain intensity has been reported previously in the literature, supporting the findings of the current study. During the development of the original scale, DASH change scores moderately correlated with changes in pain intensity (r = 0.65), consistent with our finding [2]. A translated version also demonstrated the same pattern, where changes in the Norwegian DASH showed a moderate correlation with changes in the NPRS (r = 0.69), changes in Shoulder Pain and Disability Index pain (r = 0.77) and changes in SF-36 bodily pain (r = 0.53) [21]. Similarly, an improvement in the Dutch DASH score was associated with a reduced pain intensity measured using a visual analogue scale (r = 0.55), the Constant–Murley pain domain (r = 0.45), SF-36 bodily pain (r = 0.47) and the pain domain of the five European quality of life dimensions (r = 0.41) [22].

The GRC was used in the current study as an external anchor to help in determining what was perceived as an important change. To make sure that patients’ reports using the GRC actually reflected a change in a construct similar to that measured with the DASH, i.e., upper extremity function, the wording of the GRC question was formulated to enquire about the perceived change in upper extremity function. Based on the argument that DASH change scores and the GRC reflect changes in the same construct, a hypothesized moderate positive correlation was formulated a priori. This hypothesized correlation was supported by the findings of the current study. A correlation between changes in DASH scores over time and a Global Rating of Change (7-point GRC) has been reported in patients with various upper extremity musculoskeletal disorders (r = 0.66, r = 0.72) [20,42]. In patients with shoulder disorders, the Persian and Danish DASH change scores moderately correlated (r = 0.54; r = 0.52) with a 7-point GRC [10,23], while the Norwegian DASH change scores showed a similar correlation with a 3-point GRC (r = 0.61) [21]. Furthermore, changes in DASH scores demonstrated a moderate correlation with a 15-point GRC (r = 0.60) in patients with lateral elbow tendinopathy [24]. Despite the variation in the format of the GRC in the studies discussed above, DASH change scores correlated with these GRC scales in a manner that supports the responsiveness of the DASH.

Based on the COSMIN guidelines, effect size indices by themselves are not measures of a PROM’s responsiveness; rather, they reflect the magnitude of change [7]. A specific hypothesis regarding the expected magnitude of the effect size was determined a prior in the current study. This use of the effect size as part of the specific hypothesis testing to examine responsiveness is supported according to the COSMIN guidelines [7,17]. Participants who reported an improvement in upper extremity function (based on the GRC score) were hypothesized to have at least a moderate effect size (≥0.5) given that this magnitude of change was believed to represent the clinically significant change that was expected to occur in this cohort. In the current study, a large effect size was observed in patients who improved, supporting our pre-defined hypothesis. Even when all participants in the current study were considered, the observed effect size was higher than the hypothesized magnitude of the medium effect size. During the development of the original scale, the DASH demonstrated effect sizes similar to those reported in the current study in patients receiving surgical intervention for shoulder, wrist and hand injuries with a 12-week follow-up [2]. Additionally, the Thai version of the DASH had effect sizes (SRM = 0.88 and ES  = 0.76) close to those reported in the current study [11]. When only participants who improved were considered, Schmitt et al. reported a lower magnitude of change in the DASH (SRM = 1.26 and ES = 1.21) than ours in patients who received physical therapy and occupational therapy for upper extremity musculoskeletal disorders with a 3-month follow-up [43].

After physical therapy and occupational therapy for upper extremity musculoskeletal disorders, Alotaibi et al. reported higher effect sizes (ES = 1.39, SRM = 1.51) for the Arabic DASH than those reported in the current study. This could be attributed to the shorter follow-up period employed in the current study compared to that of Alotaibi et al. (30–36 days) [9]. On the other hand, Alotaibi et al. used effect size indices to examine the responsiveness of the Arabic DASH with no pre-defined hypothesis regarding the expected magnitude and direction of change, which is considered an inappropriate method of assessing responsiveness based on the COSMIN guidelines [7,17]. A number of previous research studies reported larger effect size indices of the DASH than those reported in this study in patients who received surgical and conservative treatments [22,24,44]. These studies had longer follow-up periods than the current study, and similar to the majority of the literature, the use of effect size indices was not associated with a hypothesized magnitude and direction of these effect sizes.

The area under the ROC curve was used in the current study as part of the hypothesis testing to assess the responsiveness of the Arabic DASH. The Arabic DASH change score was able to differentiate between participants with a reported improvement in upper extremity function and those with no improvement as indicated by the AUC, which was higher than the recommended threshold of 0.7 [8,45]. This discriminatory ability of the DASH change score supports the notion that the Arabic DASH is able to detect changes in upper extremity function over time (responsiveness). The AUC reported in the current study is close to that reported by Alotaibi et al. (AUC = 0.82) for the Arabic DASH, but the 95% confidence interval of the AUC estimate was narrower in the current study, providing greater confidence in the AUC estimate [9]. The AUC reported in the current study is consistent with prior reports in the literature, such as the Italian version of the DASH (AUC = 0.87) [20], but the discriminatory ability of the Arabic DASH seems to be better than that of other versions, such as the Nepali (AUC = 0.69) [20], Dutch (AUC = 0.66) [22], Persian (AUC = 0.77) [23] and Danish versions (AUC = 0.76) [10].

The DASH MIC for improvement was determined in the current study using two methods, namely the predictive modeling method and the ROC method. Predictive modeling is believed to be the preferred method, given that it is more precise compared with the commonly used ROC method. In addition, predictive modeling allows for the inclusion of additional factors that may influence the MIC value, such as baseline status, without the need to split the sample into smaller subgroups [26]. The DASH MIC estimates determined in the current study using both the predictive modeling and ROC methods were very close. To the best of our knowledge, this is the first study that determined the MIC of the DASH using the predictive modeling method. The MIC value estimated in our study using both methods is close to that reported by Alotaibi et al. (MIC = 15) for the Arabic DASH using the ROC method, but with better diagnostic accuracy, sensitivity and specificity [9]. A change in an individual patient’s score of at least the MIC value determined in the current study (14.85) can be interpreted confidently as an important true change in the patient’s upper extremity function and could be used as a threshold for improvement to achieve during the care of individuals with upper extremity musculoskeletal disorders. This magnitude of change is a true change and not related to a measurement error, given that the MIC value exceeds the value of the Arabic DASH minimal detectable change determined previously (9.28) [9]. The MIC of the DASH reported in the current study is consistent with the estimates (MIC = 15, 20) reported during the development of the original DASH using the ROC method [2]. The DASH MIC reported in the current study falls within the MIC value range (4.4 to 25.4) reported in the literature [10,20,21,22,23,24,25,46,47]. This wide range in the literature could be attributed to the variations among the studies in participants’ characteristics, treatments received, the external anchor used and the definition of improvement vs. no improvement. A common limitation in the available studies that estimated the DASH MIC value is the unbalanced proportions of participants who improved and those who did not improve, which is known to introduce bias in the MIC estimate [27].

To the best of our knowledge, no prior studies have examined whether the MIC of the DASH is dependent on the baseline status using an appropriate methodology. Rysstad et al. reported the MIC values for patients with low and high baseline severities by subgrouping their small sample size into even smaller subgroups, which was carried out using the baseline score of the DASH [21]. Using the baseline score of the measure of interest, DASH, to split the sample into subgroups is known to cause false baseline dependence in the MIC estimates; thus, this method is not recommended and was not used in the current study [40]. An alternative method that involves using the baseline score of another PROM related to the measure of interest for stratification (low vs. high baseline severity) is recommended [40]. In line with this recommendation, the baseline NPRS score was used for stratification, given that it represents a construct related to that measured by the DASH and that it had a moderate correlation with the baseline DASH score.

The general aim of this study was to examine the responsiveness of the Arabic DASH and to quantify its MIC, and the scope of the current study was limited to patients with upper extremity musculoskeletal disorders attending physical therapy clinics in Riyadh, Saudi Arabia. Some limitations in the current study are worth mentioning. The median time interval between the baseline and follow-up assessments was relatively short; thus, some of the study findings, such as the effect size, should be interpreted by taking this time interval into account. Despite the relatively short time interval between testing sessions in some participants, about half of the participants reported an improvement in their upper extremity function. Additionally, the current study followed the COSMIN guidelines using specific pre-defined hypotheses to examine the responsiveness with an adequate sample size. This study also used the currently recommended predictive modeling method in determining the MIC and examined possible baseline dependence.

## 5. Conclusions

The aims of the current study were to examine the responsiveness of the Arabic DASH, to quantify the MIC for improvement in the Arabic DASH (using predictive modeling and ROC methods) and to examine whether the MIC value was dependent on patients’ baseline status. The Arabic DASH demonstrated sufficient responsiveness, supporting the idea that the Arabic DASH is capable of detecting changes in upper extremity function over time. The value of the Arabic DASH MIC was similar when estimated using the predictive modeling and ROC methods, and the MIC was not dependent on baseline status.

## Figures and Tables

**Table 1:** Pre-defined hypotheses to examine the responsiveness of the Arabic DASH.

Pre-Defined Hypotheses	Hypothesis Supported
1. DASH change scores demonstrate a positive correlation with GAF change scores (≥0.4).	Yes
2. DASH change scores demonstrate a positive correlation with NPRS change scores (≥0.4).	Yes
3. DASH change scores demonstrate a positive correlation with GRC scores (≥0.4).	Yes
4. The DASH demonstrates an effect size (ES, SRM) of at least 0.5 in participants with a reported improvement in upper extremity function (GRC ≥ 3).	Yes
5. DASH change scores differentiate between participants with a reported improvement in upper extremity function and those with no improvement (area under the ROC curve (AUC) ≥ 0.70).	Yes

DASH = Disabilities of the Arm, Shoulder and Hand; GAF = Global Assessment of Function; NPRS = Numeric Pain Rating Scale; GRC = Global Rating of Change Scale; ES = effect size; SRM = standardized response mean; ROC = receiver operating characteristic; AUC = area under the curve.

**Table 2:** Characteristics of participants (*N* = 115).

Variable	Mean ± SD or *N* (%)
Age (year)	38.18 ± 13.98
Sex	
Male	72 (62.6)
Female	43 (37.4)
Height (m)	1.68 ± 0.09
Mass (kg)	76.39 ±16.56
Body mass index (kg/m^2^)	27.26 ± 5.99
Site of dysfunction	
Shoulder and arm	50 (43.5)
Elbow and forearm	21 (18.3)
Wrist and hand	44 (38.3)
Upper extremity surgery	
Yes	51 (44.3)
Time after surgery (months)	1.84 (2.76) *
No	64 (55.7)
Duration of symptoms (months)	2.99 (7.71) *

* = median (interquartile range).

**Table 3:** Outcome measures at baseline and follow-up.

Variable	Baseline Scores Mean ± SD	Follow-Up Scores Mean ± SD	Change Scores Mean ± SD	ES	SRM	Baseline		Follow-Up	
						Floor	Ceiling	Floor	Ceiling
DASH (0–100)	47.47 ± 21.17	30.33 ± 23.66	17.14 ± 24.73	0.81	0.69	0%	0%	0%	10.4%
Improved (*N* = 56)	52.52 ± 21.50	19.53 ± 20.46	32.99 ± 23.23	1.53	1.42				
Unchanged (*N* = 58)	42.25 ± 19.77	40.22 ± 22.03	2.03 ± 14.88	0.10	0.14				
GAF (0–100)	58.70 ± 19.65	75.83 ± 20.74	17.14 ± 22.16	0.87	0.77	0%	0%	0%	12.2%
NPRS (0–10)	5.00 ± 2.41	3.17 ± 2.81	1.84 ± 2.90	0.76	0.63	2.6%	3.5%	1.7%	25.2%

ES = effect size; SRM = standardized response mean; DASH = Disabilities of the Arm, Shoulder and Hand; GAF = Global Assessment of Function; NPRS = Numeric Pain Rating Scale. Floor represents the percentage of participants with the worst score (worst status), while ceiling represents the percentage of participants with the best score (best status).

**Table 4:** Participants according to their global rating of change score at follow-up.

Variable	*N* (%)
GRC	
5 (Very great deal better)	25 (21.7)
4 (Great deal better)	15 (13.0)
3 (Moderately better)	16 (13.9)
2 (Little bit better)	26 (22.6)
1 (A tiny bit better, almost the same)	14 (12.2)
0 (No change)	13 (11.3)
−1 (Tiny bit worse, almost the same)	5 (4.3)
−2 (Little bit worse)	0 (0.0)
−3 (Moderately worse)	1 (0.9)
−4 (Great deal worse)	0 (0.0)
−5 (Very great deal worse)	0 (0.0)
Change over time status according to GRC score	
Unchanged vs. Improved vs. Worsened	
Unchanged (GRC −2 to 2)	58 (50.4)
Improved (GRC ≥ 3)	56 (48.7)
Worsened (GRC ≤ -3)	1 (0.9)
Improved vs. Not improved	
Improved (GRC ≥ 3)	56 (48.7)
Not improved (GRC ≤ 2)	59 (51.3)

GRC = Global Rating of Change Scale.

**Table 5:** Correlation between the DASH change score and change in other measures.

Variable	r (95% CI)	*p*
GAF change	0.69 (0.55 to 0.81)	<0.001
NPRS change	0.68 (0.57 to 0.77)	<0.001
GRC	0.73 (0.64 to 0.80) *	<0.001

r = Pearson’s correlation coefficient; CI = confidence interval; DASH = Disabilities of the Arm, Shoulder and Hand; GAF = Global Assessment of Function; NPRS = Numeric Pain Rating Scale; GRC = Global Rating of Change Scale. * Spearman’s correlation coefficient.

## Data Availability

The data presented in this study are available from the corresponding author on reasonable request.
